# Supplementary material for: Iterative improvement in the automatic modular design of robot swarms
Source: PeerJ Comput Sci. 2020 Dec 7;6:e322. doi: 10.7717/peerj-cs.322 (PMC7924708; doi:10.7717/peerj-cs.322)
Supplement: Supplemental Information 3 [file peerj-cs-06-322-s003.zip › argos3/doc/api/standalone/a00407_source.html]

ARGoS: core/utility/string\_utilities.h Source File


- Main Page
- Related Pages
- Namespaces
- Classes
- Files

- File List
- File Members

# core/utility/string\_utilities.h

Go to the documentation of this file.

```
00001 
00009 #ifndef STRING_UTILITIES_H
00010 #define STRING_UTILITIES_H
00011 
00012 #include <argos3/core/utility/configuration/argos_exception.h>
00013 #include <argos3/core/utility/datatypes/datatypes.h>
00014 #include <string>
00015 #include <vector>
00016 #include <sstream>
00017 
00018 namespace argos {
00019 
00020    /****************************************/
00021    /****************************************/
00022 
00036    template<typename T> std::string ToString(const T& t_value) {
00037       std::ostringstream ss;
00038       ss.setf(std::ios::boolalpha);
00039       ss << t_value;
00040       return ss.str();
00041    }
00042 
00043    /****************************************/
00044    /****************************************/
00045 
00059    template<typename T> T FromString(const std::string& str_value) {
00060       T tReturnValue;
00061       std::istringstream ss(str_value);
00062       ss.setf(std::ios::boolalpha);
00063       ss >> tReturnValue;
00064       return tReturnValue;
00065    }
00066 
00067    /****************************************/
00068    /****************************************/
00069 
00070    template<typename T> void ParseValues(std::istream& str_input,
00071                                          UInt32 un_num_fields,
00072                                          T* pt_field_buffer,
00073                                          const char ch_delimiter = '\n') {
00074       std::vector<std::string> s(un_num_fields);
00075       UInt32 i = 0;
00076       while(i < un_num_fields && std::getline(str_input, s[i], ch_delimiter)) {
00077          i++;
00078       }
00079       if (i == un_num_fields) {
00080          str_input.clear(); // the istream was read completely and this is fine, so set the flag to 'good'
00081          for(i = 0; i < un_num_fields; i++) {
00082             std::istringstream iss(s[i]);
00083             iss >> pt_field_buffer[i];
00084          }
00085       }
00086       else {
00087          THROW_ARGOSEXCEPTION("Parse error: expected " << un_num_fields
00088                               << " values, but " << i << " have been found in \""
00089                               << str_input.rdbuf() << "\"");
00090       }
00091    }
00092 
00093    /****************************************/
00094    /****************************************/
00095 
00096    template<typename T> void ParseValues(const std::string& str_input,
00097                                          const UInt32 un_num_fields,
00098                                          T* pt_field_buffer,
00099                                          const char ch_delimiter = '\n') {
00100       std::istringstream issInput(str_input);
00101       ParseValues(issInput, un_num_fields, pt_field_buffer, ch_delimiter);
00102    }
00103 
00104    /****************************************/
00105    /****************************************/
00106 
00113       void Tokenize(const std::string& str_string,
00114                     std::vector<std::string>& vec_tokens,
00115                     const std::string& str_delimiters = " ");
00116 
00122       std::string StringToUpperCase(const std::string& str_string);
00123 
00129       std::string StringToLowerCase(const std::string& str_string);
00130 
00131       /****************************************/
00132       /****************************************/
00133 
00141       void Replace(std::string& str_buffer,
00142                    const std::string& str_original,
00143                    const std::string& str_new);
00144 
00145       /****************************************/
00146       /****************************************/
00147 
00155       bool MatchPattern(const std::string& str_input,
00156                         const std::string& str_pattern);
00157 
00158       /****************************************/
00159       /****************************************/
00160 
00167       std::string& ExpandEnvVariables(std::string& str_buffer);
00168 
00169       /****************************************/
00170       /****************************************/
00171 
00172 }
00173 
00174 #endif
```

---

Generated on 10 Jul 2018 for ARGoS by 
 1.6.1
